# FX11 limits *Mycobacterium tuberculosis* growth and potentiates bactericidal activity of isoniazid through host-directed activity

**DOI:** 10.1242/dmm.041954

**Published:** 2020-03-30

**Authors:** Gopinath Krishnamoorthy, Peggy Kaiser, Ulrike Abu Abed, January Weiner, Pedro Moura-Alves, Volker Brinkmann, Stefan H. E. Kaufmann

**Affiliations:** 1 Department of Immunology, Max Planck Institute for Infection Biology, Berlin 10117, Germany; 2 Core Facility Microscopy, Max Planck Institute for Infection Biology, Berlin 10117, Germany; 3 Ludwig Institute for Cancer Research, Nuffield Department of Clinical Medicine, University of Oxford, Oxford OX3 7DQ, UK; 4 Hagler Institute for Advanced Study at Texas A&M University, College Station, TX 77843-3572, USA

**Keywords:** Glycolysis, Lactate dehydrogenase A, FX11, *Mycobacterium tuberculosis*, Granuloma, Hypoxia, Immunometabolism, Host-directed therapy

## Abstract

Lactate dehydrogenase A (LDHA) mediates interconversion of pyruvate and lactate, and increased lactate turnover is exhibited by malignant and infected immune cells. Hypoxic lung granuloma in *Mycobacterium tuberculosis*-infected animals present elevated levels of *Ldha* and lactate. Such alterations in the metabolic milieu could influence the outcome of host-*M. tuberculosis* interactions. Given the central role of LDHA for tumorigenicity, targeting lactate metabolism is a promising approach for cancer therapy. Here, we sought to determine the importance of LDHA for tuberculosis (TB) disease progression and its potential as a target for host-directed therapy. To this end, we orally administered FX11, a known small-molecule NADH-competitive LDHA inhibitor, to *M. tuberculosis*-infected C57BL/6J mice and *Nos2*^−/−^ mice with hypoxic necrotizing lung TB lesions. FX11 did not inhibit *M. tuberculosis* growth in aerobic/hypoxic liquid culture, but modestly reduced the pulmonary bacterial burden in C57BL/6J mice. Intriguingly, FX11 administration limited *M. tuberculosis* replication and onset of necrotic lung lesions in *Nos2*^−/−^ mice. In this model, isoniazid (INH) monotherapy has been known to exhibit biphasic killing kinetics owing to the probable selection of an INH-tolerant bacterial subpopulation. However, adjunct FX11 treatment corrected this adverse effect and resulted in sustained bactericidal activity of INH against *M. tuberculosis*. As a limitation, LDHA inhibition as an underlying cause of FX11-mediated effect could not be established as the on-target effect of FX11 *in vivo* was unconfirmed. Nevertheless, this proof-of-concept study encourages further investigation on the underlying mechanisms of LDHA inhibition and its significance in TB pathogenesis.

## Abstract (precis)

**Summary:** Targeting LDHA results in tumor regression in experimental models. Likewise, co-administration of a known small molecule LDHA inhibitor and an anti-tuberculosis drug has improved the tuberculosis therapeutic outcomes in murine models.

## INTRODUCTION

Tuberculosis (TB) is the leading cause of mortality from a single infectious agent globally (WHO, 2018) and its treatment includes 6-month-long therapy with combinations of drugs. Inappropriate treatment or non-compliance results in emergence of multi-drug-resistant TB (MDR-TB), which renders current treatment options ineffective. Development of newer drugs with superior efficacy and safety is urgently required to shorten the treatment duration as well as to manage MDR-TB effectively. Although pathogen-targeted treatment is the preferred choice, adjunct therapeutics directed at the host immune system are being increasingly recognized for their potential to reduce pathogen load and ameliorate exacerbated organ damage during TB disease progression (Costa et al., 2016; Dorhoi and Kaufmann, 2016; Frank et al., 2018; Hawn et al., 2013; Kaufmann et al., 2018; Matty et al., 2019; Olive and Sassetti, 2016; Russell et al., 2019; Singhal et al., 2014; Subbian et al., 2016; Tiberi et al., 2018). Radiotracer imaging has revealed heterogeneity – in size, metabolism and infection – within and between granulomas in a single host infected with *Mycobacterium* *tuberculosis* (Lenaerts et al., 2015; Lin et al., 2014). In general, the impact of metabolic pathways (such as glycolysis) and mitochondrial respiration on immune functions and host-pathogen interactions is increasingly accepted (Eisenreich et al., 2017; Escoll and Buchrieser, 2018; Escoll et al., 2017; Kiran et al., 2016; Olive and Sassetti, 2016; Russell et al., 2019). Heterogeneous responses in granuloma, therefore, could partly be attributed to metabolic state(s)/energy phenotype(s) of different immune cells (e.g. macrophages, neutrophils, lymphocytes) that are influenced by their microenvironment and local infection dynamics. Understanding of pathogen-induced immunometabolic dysregulation in granuloma can provide insights into the vital pathways in the infected host and thereby reveal novel therapeutic target candidates.

Untargeted metabolite analysis has identified elevated levels of lactate in necrotic granuloma of *M. tuberculosis*-infected guinea pigs (Somashekar et al., 2011). Generation of lactate from pyruvate in hypoxic cells is catalyzed by lactate dehydrogenase A (LDHA), the functions of which depend on hypoxia-inducible factors (HIFs) (Gordan et al., 2007). Both *Ldha* and *Hif1a* transcripts have been found to be significantly induced during early stages of granuloma formation in a murine model (Domingo-Gonzalez et al., 2017; Shi et al., 2015), and the essential function of HIF1α in controlling TB progression has already been recognized (Braverman et al., 2016). Although metabolic phenotypes of malignant and immune cells show some critical differences, they present many similarities (Andrejeva and Rathmell, 2017). In most cancer cells, aerobic glycolysis (Warburg effect), or hypoxia adaptation, requires LDHA, and its inactivation using the NADH-competitive inhibitor 3-dihydroxy-6-methyl-7-(phenylmethyl)-4-propylnaphthalene-1-carboxylic acid (FX11; PubChem CID: 10498042), or transcriptional repression, has been shown to cause regression of lymphoma and pancreatic cancer (Fantin et al., 2006; Le et al., 2010). In this report, we examined whether administering FX11 could result in host-beneficial and pathogen-detrimental outcome in murine TB models and its relevance to host-directed therapy of this devastating disease.

## RESULTS

**Fig. 1.**
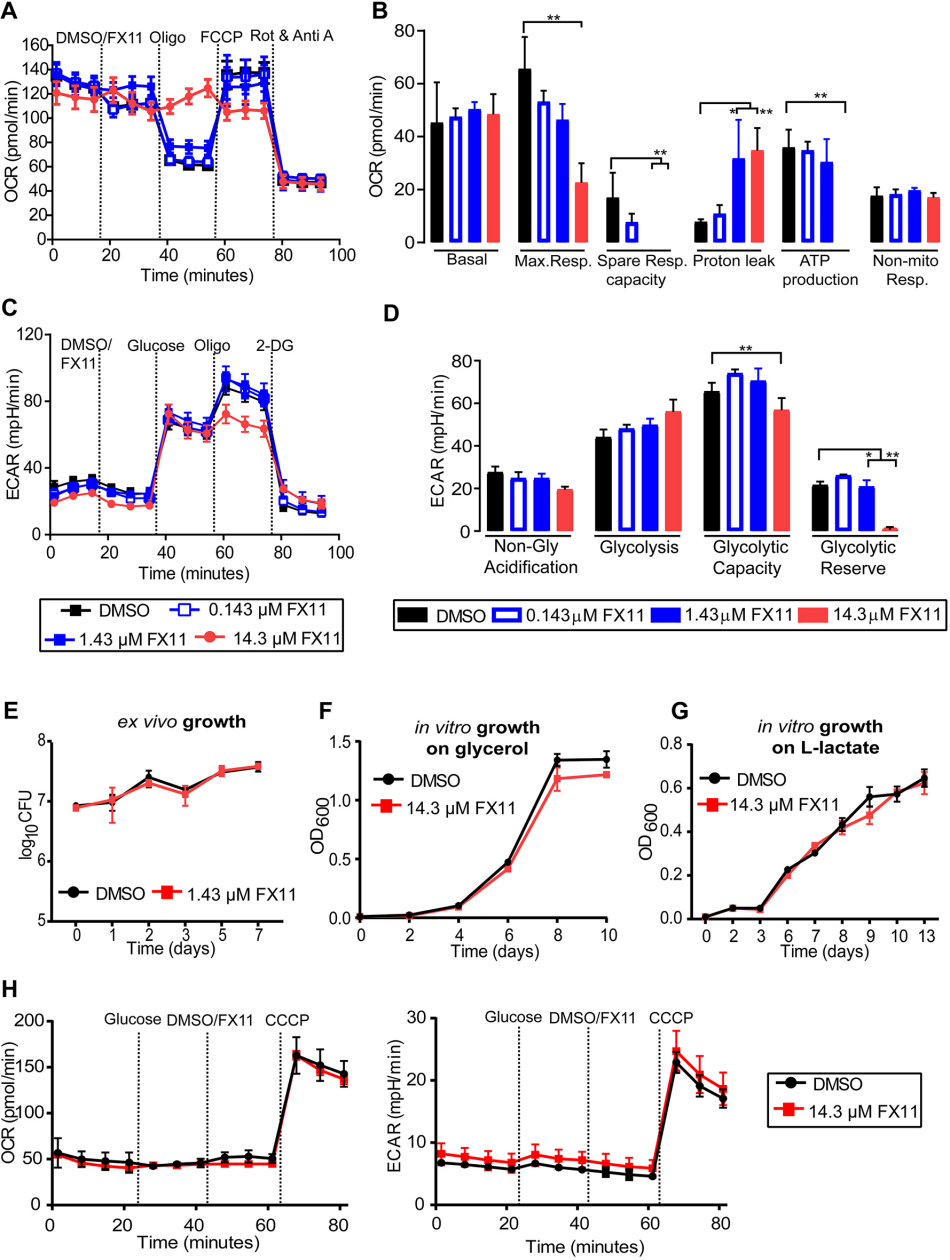
**FX11-induced metabolic changes are highly host specific.** (A-D) FX11 alters the respiratory profile and parameters (A,B), and glycolytic parameters (C,D) of IFN-γ-stimulated murine bone marrow-derived macrophages (BMDMs) in a concentration-dependent manner. Wells with DMSO served as a control. Different mitochondrial and glycolytic modulators were sequentially injected and cellular responses (OCR and ECAR values) were measured using a Seahorse XF analyzer. The error bars are standard deviations of the data from three independent experiments. Statistical significance was determined by Student's *t*-test for each of the concentrations, compared to the DMSO control. Adjusted *P*-values were corrected for multiple testing using the Benjamini-Hochberg correction, as indicated. *P<0.001, **P<0.0001. In addition, linear regression analysis was carried out to independently determine the statistical significance (see Supplementary Materials and Methods). (E) IFN-γ-stimulated BMDMs infected with *M. tuberculosis* H37Rv at a multiplicity of infection of 1:5, with FX11 effect determined by enumerating viable bacterial counts. (F,G) Effect of FX11 on *M. tuberculosis* growth in liquid medium containing 0.2% v/v glycerol (F) or 10 mM sodium L-lactate (G) as the sole carbon source. (H) Effect of FX11 on *M. tuberculosis* respiratory function [OCR (left) and ECAR (right) values] measured by Seahorse XFp extracellular flux analyzer. DMSO, dimethyl sulfoxide; ECAR, extracellular acidification rate; FCCP, carbonyl cyanide-4-(trifluoromethoxy)phenylhydrazone; OCR, oxygen consumption rate; OD_600_, optical density at a wavelength of 600 nm; Rot & Anti A, Rotenone and Antimycin A; 2-DG, 2-deoxy-D-glucose.

Inhibition of LDHA with FX11 reduces mitochondrial membrane potential and inhibits glycolysis in human Panc (P) 493 B-lymphoid cells (Le et al., 2010). We assessed the FX11-induced effect in interferon-gamma (IFN-γ)-stimulated, but uninfected, murine bone marrow-derived macrophages (BMDMs). FX11 addition increased the oxygen consumption rate (OCR), but decreased the respiratory capacity and ATP synthesis (Fig. 1A,B; Supplementary Materials and Methods). Essentially, FX11, at 14.3 µM concentration, uncoupled the mitochondrial respiratory chain from the phosphorylation system. However, FX11 addition had less impact on glycolysis in BMDMs, although it depleted the cellular glycolytic reserve at highest concentration (Fig. 1C,D). These observations, therefore, confirm that FX11 primarily affects mitochondrial energy generation in BMDMs by inhibiting LDHA function, as reported by others (Fantin et al., 2006; Le et al., 2010; Sonveaux et al., 2008).

Intriguingly, recent studies have demonstrated that energy flux changes – in aerobic glycolysis and fatty acid oxidation – are dependent on the virulence and viability of *M. tuberculosis* or host cellular types (Braverman et al., 2016; Cumming et al., 2018; Gleeson et al., 2016; Lachmandas et al., 2016). Consequently, we examined whether FX11-mediated impairment of respiratory/glycolytic function directly affects the intramacrophage survival of *M. tuberculosis*. As high concentration of FX11 affected the long-term viability of BMDMs, we tested a low concentration of 1.43 µM and found that bacterial survival remained comparable between untreated and FX11-treated conditions (Fig. 1E). FX11 is an analog of anti-bacterial gossypol, a potent inhibitor of LDHA (Margalith, 1967). Therefore, we assessed the effect of FX11 (14.3 µM) on *M. tuberculosis* axenic culture. Our results, however, showed that FX11 did not affect the aerobic growth of *M. tuberculosis* in glycerol or sodium L-lactate as a sole carbon source, when compared with the untreated growth control (Fig. 1F,G). This effect was validated further using *M. tuberculosis* expressing green fluorescent protein. Fluorescence measurement during the course of incubation under normoxia and hypoxia (1% O_2_), as a function of growth, revealed that FX11 did not affect bacterial viability, although a minor decrement in fluorescence was noted (Fig. S1A,B). Moreover, the development of a pale brownish color was noted in FX11-supplemented hypoxic culture, suggesting that this small molecule is differentially metabolized under such condition. Finally, the respiratory functions in *M. tuberculosis* also remained unperturbed when FX11 was added (Fig. 1H). Taken together, these results indicate that the bioenergetic effects of FX11 are highly host cell specific.

**Fig. 2.**
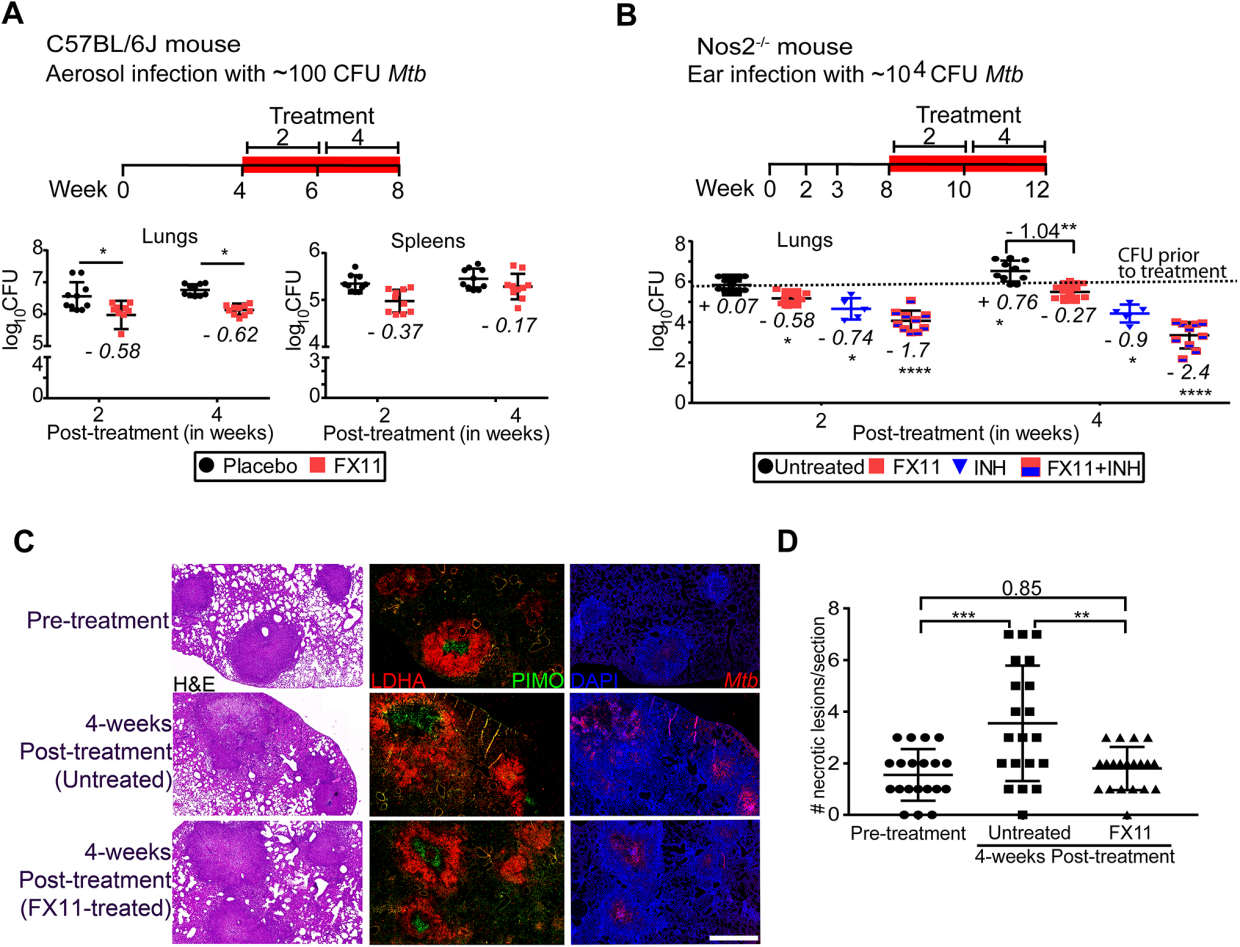
**FX11 effects on *M. tuberculosis* in mouse models.** (A) Schematic representation of experimental design (treatment duration is highlighted in red). Effect of FX11 (2 mg/kg) on bacterial burden in C57BL/6J mice aerosol infected with 100 CFU *M. tuberculosis*. Datasets presented are from two independent experiments (total *n*=10). Values shown are means±s.d. Italicized numerical value (negative) represents reduction in log_10_ CFU in the treated group, when compared with the placebo control group. Statistical significance was evaluated using an unpaired Student’s *t-*test. *P<0.05. (B) Effect of FX11 (2 mg/kg) as a monotherapy or in combination with isoniazid (INH) (25 mg/kg) in *Nos2*^−/−^ mice with hypoxic necrotizing lung lesions (Gengenbacher et al., 2017). The TNFα response was neutralized at 2 and 3 weeks post-infection. Drugs were administered after onset of central necrosis and hypoxia in lung lesions on day 56. Untreated or INH-treated groups were used for comparisons. Lung CFU data (means±s.d.) from two independent experiments (total *n*=9-10) are shown. Lung CFU data (means±s.d.) of INH-treated group are from a single experiment with a group size of 5. Italicized numerical value (negative) represents reduction or value (in positive) represents a further increase in log_10_ CFU in the specified group, when compared with the control group prior to drug treatment (i.e. day 56, indicated with a dotted line). Pooled data from two independent experiments were analyzed using nonparametric Mann–Whitney test (data that did not pass the Shapiro–Wilk normality test). Statistical significance as compared to the group prior to drug treatment, *P<0.05, **P<0.01, ****P<0.0001. (C) Hematoxylin and Eosin (H&E) staining and immunofluorescence detection of *M. tuberculosis* or hypoxia marker pimonidazole (PIMO) and LDHA. Magnified images show the staining of LDHA and PIMO in a lung lesion. Scale bar: 1 mm. Micrographs of a stained section of whole left lung lobe are presented in Fig. S3. (D) Total numbers (means±s.d.) of necrotizing lesions present in *Nos2*^−/−^ mice that were either untreated or FX11 treated. Data from a representative experiment (total *n*=5) were analyzed using two-way ANOVA with multiple comparison and Tukey's post-test. Statistical significance as compared to the control group prior to drug treatment, **P<0.01, ***P<0.001.

Subsequently, the effect of FX11 was evaluated in two murine TB models. In the initial experiment, C57BL/6J mice were aerosol infected with ∼100 colony-forming units (CFU) of *M. tuberculosis* H37Rv. At 4 weeks post-infection, mice received either dimethyl sulfoxide (DMSO)-solubilized FX11 (2 mg/kg) or 2% DMSO (final concentration) as placebo by oral gavage (6 days/week) for a further 4 weeks. The administered dose of FX11 is similar to that in a previous study (Le et al., 2010), and further dose increment is restricted due to poor compound solubility. Post-treatment effect was monitored at 2 and 4 weeks by enumerating CFU from excised lungs and spleens of euthanized animals. FX11 administration resulted in approximately 0.5 log_10_ reduction in pulmonary *M. tuberculosis* counts (Fig. 2A; Fig. S2A) with less apparent effect on splenic bacterial load.

TB lesions in C57BL/6J mice rarely progress into necrosis and lack reliability in predicting the efficacy of anti-TB drug in humans. We intend to generate proof-of-concept evidence using a *Nos2*^−/−^ mouse model [after neutralization of tumor necrosis factor alpha (TNFα)], which presents hypoxic necrotizing lung lesions and has been found reliable for pre-clinical testing of several anti-TB drugs (Duque-Correa et al., 2014; Gengenbacher et al., 2017; Reece et al., 2010). In addition, in this mouse model, isoniazid (INH) has been reported to exhibit bi-phasic bactericidal effect – similar to the guinea pig model (Ahmad et al., 2009) – which has been correlated with necrotic lesions, a niche permitting the evolution of slow/non-growing drug-tolerant subpopulation of *M. tuberculosis* (Gengenbacher et al., 2017). Therefore, in the subsequent experiment, the effect of FX11 (2 mg/kg), either alone or in combination with INH (25 mg/kg), was evaluated in *Nos2*^−/−^ mice (Fig. 2B). Efficacy was determined by assessing bacterial viability and histopathology. FX11 administration was apparently well tolerated because treated animals showed no increased distress or weight loss (Fig. S2B). FX11 treatment alone failed to significantly reduce the bacterial burden in the organs, but restricted further bacterial replication in the lung, when compared with the untreated control group. Most notably, the combination of FX11 and INH resulted in better efficacy, and there was no further cessation of bactericidal activity of INH, when compared with monotherapy (Fig. 2B). As previously observed (Gengenbacher et al., 2017), onset of pimonidazole (PIMO)-stained hypoxic lesions became apparent on day 56 (at treatment start). Although the number and size of lesions were comparable, further development of necrotic lesions ceased in the FX11-treated group (Fig. 2C,D; Fig. S2C,D). Such FX11-mediated inhibition of progression to necrosis could have potentiated the efficacy of INH by preventing the emergence of the drug-tolerant population. However, this hypothesis requires further validation using other mouse models that develop necrotic (C3HeB/FeJ) or non-necrotic lesions (C57BL/6J or BALB/c) upon *M. tuberculosis* infection. Furthermore, immunofluorescence staining of paraffin-embedded lung sections revealed that LDHA expression is predominantly surrounding the lesions (Fig. 2C; Fig. S3), indicating its relevance. Nonetheless, FX11 administration had no apparent impact on LDHA abundance as measured by immunofluorescence. In this case, as an NADH-competitive inhibitor, FX11 affects only the LDHA enzymatic turnover and its biochemical function and any suppression on transcription/translation level is unlikely. Furthermore, our attempts to quantify lactate, using an enzymatic method, from the excised whole-lung tissues of FX11-treated animals presented results with poor reproducibility, which were difficult to interpret reliably. Although, FX11 is a well-known inhibitor of LDHA (Deck et al., 1998; Le et al., 2010; Zhang et al., 2015), owing to the lack of evidence, LDHA inhibition mediated by FX11 is unsubstantiated.

## DISCUSSION

Lactate regulates key processes in tumor progression and tumor immunity (Brand et al., 2016; Haas et al., 2015; Sonveaux et al., 2008). Likewise, lactate metabolism is assumed to affect major features of the host immune response, *M. tuberculosis* adaptation and host-pathogen interactions (Billig et al., 2017; Braverman et al., 2016; Gleeson et al., 2016; Lachmandas et al., 2016; Qualls and Murray, 2016; Serafini et al., 2019; Shi et al., 2016, 2015; Somashekar et al., 2011). In this report, FX11, a known direct competitive inhibitor of murine LDHA, ameliorated disease pathology, restricted mycobacterial growth and potentiated the bactericidal effect of INH, a frontline anti-TB drug. Our observations are intriguing and consistent with those reported in other studies, wherein the pharmacological inhibition of host immune function has been shown to potentiate the efficacy of anti-TB drugs and has been implicated as an adjunct host-directed therapy with the potential to improve treatment outcome (Cheng et al., 2017; Costa et al., 2016; Dutta et al., 2007; Gupta et al., 2017; Maiga et al., 2015; Singhal et al., 2014; Subbian et al., 2016, 2011; Xu et al., 2018). However, the observed FX11-mediated effect requires further validation in C3HeB/FeJ mice that develop hypoxic necrotic granuloma, because the impaired nitric oxide production in immune cells of *Nos2*^−/−^ mice could have confounding effects.

FX11 is a highly specific inhibitor of LDHA and has been previously used for LDHA depletion in *in vivo* mouse and in *in vitro* experimental models (Im et al., 2019; Le et al., 2010; Rajeshkumar et al., 2015; Xian et al., 2015; Zhang et al., 2015). However, our results were inconclusive in confirming the on-target effect and it is not clear whether LDHA inhibition is an underlying cause of FX11-mediated effect. Furthermore, the impact of LDHA inhibition on immune responses to infection remains poorly studied. Perhaps the advances in tumor immunometabolism could be useful to infer plausible mechanism(s) associated with LDHA inhibition in the context of TB pathogenesis. First, LDHA depletion has been shown to impede progression of necrosis in tumors, which strongly suggests its essential role in pathophysiology (Serganova et al., 2018). Moreover, a probable link between lactate metabolism and IFN-γ-dependent tumor immunity has been increasingly recognized. Lactate accumulation has been indicated to severely impair IFN-γ-dependent tumor immunosurveillance (Brand et al., 2016). LDHA transcriptionally regulates IFN-γ expression in T cells, and genetic ablation of LDHA in T cells has been found to protect mice from lethal IFN-γ-dependent immunopathology (Peng et al., 2016). Besides, HIF-1α is not only a transcriptional regulator of LDHA, but also coordinates components of cancer development and progression and anti-tumor immunity (Gordan et al., 2007; Serganova et al., 2018). Note that rather similar events occur in immune cells infected with *M. tuberculosis* (Braverman et al., 2016; Gleeson et al., 2016; Lachmandas et al., 2016; Serafini et al., 2019; Shi et al., 2015; Somashekar et al., 2011). Furthermore, HIF-1α has been shown to regulate IFN-γ-dependent adaptive immunity to *M. tuberculosis* (Braverman et al., 2016). It is a well-established paradigm that IFN-γ mediates a central role in macrophage and neutrophil functions and in tissue protection in TB (Desvignes and Ernst, 2009; Dorhoi et al., 2014; Mishra et al., 2013; Nandi and Behar, 2011; Pagán and Ramakrishnan, 2018). IL-17 (also known as IL17A)-dependent repression of HIF1α (and lactate accumulation) as well as hypoxic necrotic granuloma development have been reported in C3HeB/FeJ mice infected with an *M. tuberculosis* clinical isolate (Domingo-Gonzalez et al., 2017). Thus, it appears that the impact of LDHA inhibition on anti-tumor and anti-tubercular immunity may have some similarities.

Our results showed that FX11 disrupts mitochondrial functions and depletes the glycolytic reserve in murine BMDMs. Such bioenergetics changes are in agreement with several findings that examined the impact of LDHA depletion in tumor cells by FX11 inhibition or *LDHA* gene knockdown (Fantin et al., 2006; Le et al., 2010; Sonveaux et al., 2008). However, the impact of FX11 on glycolysis in BMDMs was only marginal in contrast to that in human B-lymphoid cells (Le et al., 2010). Moreover, it was shown that the LDHA-dependent glycolytic tumor cells are increasingly susceptible to FX11 treatment, when compared with other cells dependent on mitochondrial oxidative phosphorylation for energy generation (Le et al., 2010). Therefore, diverse cellular types – with distinct energy metabolic state – in granuloma may respond differently to FX11-mediated LDHA inhibition. Moreover, broad cellular activity of FX11 is undesirable; rather a cell-specific genetic ablation of *LDHA* could be appropriate for dissecting underlying immune mechanisms. In addition, there are few theoretical possibilities for potential off-target effects of FX11. Le et al. (2010) suggested that the reactive catechol moiety of FX11 or its drug intermediates (under oxygen-limiting conditions) could cause pleiotropic effects. Likewise, FX11 has been shown to induce oxidative stress, which could restrict bacterial growth and augment INH efficacy against *M. tuberculosis*. Finally, FX11 administration could deprive *M. tuberculosis* from utilizing host-derived lactate for energy generation (Billig et al., 2017; Serafini et al., 2019). In conclusion, this proof-of-concept study encourages further *in vivo* investigation to understand the role of LDHA in TB pathogenesis and its potential as a target for host-directed therapy.

## MATERIALS AND METHODS

### Bacterial strains

*M. tuberculosis* H37Rv (American Type Culture Collection, #27294) or its derivative expressing pGFPHYG2 replicative plasmid (Addgene #30173; deposited by Lalita Ramakrishnan; Cosma et al., 2006) was grown in Middlebrook 7H9 broth (Becton Dickinson) supplemented with albumin-dextrose-catalase enrichment (Becton Dickinson), 0.2% glycerol, 0.05% Tween 80, or on Middlebrook 7H11 agar (Becton Dickinson) containing 10% v/v oleic acid-albumin-dextrose-catalase enrichment (Becton Dickinson) and 0.2% glycerol. Ten milligrams of FX11 (Merck Millipore) were dissolved in 1 ml of DMSO.

### Growth assay

Bacterial growth (with 5% DMSO or 14.3 µM FX11) was assessed in minimal medium (0.5 g/l asparagine, 1 g/l KH_2_PO_4_, 2.5 g/l Na_2_HPO_4_, 50 mg/l ferric ammonium citrate, 0.5 g/l magnesium sulfate, 0.5 mg/l calcium chloride and 0.1 mg/l zinc sulfate) containing either 0.2% glycerol (v/v), or 10 mM sodium L-lactate. Cell densities (OD) were measured at 600 nm using a cell density meter (BioChrome Biowave). *M. tuberculosis* expressing green fluorescent protein (inoculum OD_600_=0.1) was cultured in minimal medium containing either 0.2% glycerol (v/v) or 0.5% glucose (w/v), 0.01% cholesterol (w/v), 10 mM sodium L-lactate with 5% DMSO or 14.3 µM FX11 in an orbital shaking incubator (normoxia), or in an anaerobic chamber supplied with 1% oxygen and 5% CO_2_. At indicated time points, 200 µl of culture was aliquoted into black, optical-bottom, 96-well microplates and fluorescence measured with a GloMax^®^ Microplate Multimode Reader using ‘Blue’ filter [excitation (Ex): 490 nm; emission (Em): 510–570 nm].

Infection stocks were prepared from mid-log phase *M. tuberculosis* cultures. For CFU determinations, serial dilutions were performed in PBS/0.05% Tween 80 and plated onto Middlebrook 7H11 agar. Plates were incubated at 37°C for 4–5 weeks prior to CFU counting.

### Drugs, formulations and administration

FX11 (Merck Millipore) or INH (Sigma-Aldrich) were formulated in 0.4% methylcellulose. The final concentration of DMSO did not exceed 2%. Drug formulations were prepared every week and stored at 4°C. Drugs were administered by oral gavage (0.2 ml) 6 days per week.

### Ethical statement

All animal studies have been ethically reviewed and approved by the State Office for Health and Social Services, Berlin, Germany. Experimental procedures were carried out in accordance with the European directive 2010/63/EU on Care, Welfare and Treatment of Animals.

### Animal experiments

Female C5BL/6J and C57BL/6J *Nos2*^−/−^ mice were bred in-house and maintained under specific pathogen-free conditions. C5BL/6J mice (aged 6–8 weeks) were aerosol infected with 100 CFU *M. tuberculosis* H37Rv. C5BL/6J *Nos2*^−/−^ mice were infected as previously reported (Gengenbacher et al., 2017). In brief, 6- to 8-week-old female C5BL/6J *Nos2*^−/−^ mice were anesthetized (ketamine 65 mg/kg, acepromazine 2 mg/kg, xylazine 11 mg/kg) and infected with 1000 CFU of *M. tuberculosis* in 20 μl PBS administered to the ear dermis. At 14 and 21 days post-infection, each mouse received 0.5 mg of monoclonal anti-TNFα antibody (purified from MP6-XT22 cultures) by intraperitoneal injection. Two hours before euthanasia, animals received 60 mg/kg pimonidazole hydrochloride (Hypoxyprobe™-1, Burlington, MA, USA) intraperitoneally to allow for detection of hypoxic regions in organ sections.

### Staining procedures and histopathology

The left lung lobe of mice was removed aseptically and post-fixed in 4% paraformaldehyde for 16–20 h at room temperature. The tissue was then dehydrated and paraffin embedded (60°C) using a Leica TP 1020 tissue processor. Paraffin blocks were cut at 2–3 μm, and sections were mounted and dried on Superfrost Plus slides (Thermo Fisher Scientific), avoiding temperatures above 37°C. After dewaxing and rehydration, sections were subjected to Hematoxylin and Eosin (H&E) staining, or fluorescence staining, to detect LDHA expression, PIMO and *M. tuberculosis* in tissues. Sections were stained with H&E using standard protocols. Central necrosis of lesions was defined as a lighter pink region, indicating tissue consolidation, surrounded by granulomatous inflammatory infiltrates. A researcher blinded to the study groups scored at least four individual stained sections of each organ in study groups of five mice per time point.

For immunostaining, sections were incubated in heat-induced epitope retrieval (HIER) buffers (pH 6, 10 mM citrate; Dako S236984-3) for 20 min at 96°C in a steam cooker (Braun). After antigen retrieval, sections were left in the same HIER buffer at room temperature to cool below 30°C. Sections were further rinsed three times with deionized water and once with Tris-buffered saline [TBS; Pierce Protein-Free Blocking Buffer (pH 7.4)]. Subsequently, sections were permeabilized for 5 min with 0.5% Triton X-100 in TBS at room temperature, followed by three rinsing steps with TBS. Slide-mounted tissue sections were encircled with a hydrophobic barrier using PAP pen (Z672548, Sigma-Aldrich) and subsequently treated with TBS blocking buffer (TBS supplemented with 1% bovine serum albumin, 2% normal donkey serum, 5% cold water fish gelatin, 0.05% Tween 20, 0.05% Triton X-100) for 30 min to prevent non-specific binding. Primary antibodies were diluted in TBS blocking buffer and incubated on the sections overnight at room temperature.

The following antibodies were used for immunostaining: rabbit anti-*M. tuberculosis* antibody (Abcam, ab905; 1:1000) and rabbit, anti-LDHA antibody (Abcam, ab101562, LOT GR176934; 1:100), with secondary detection with donkey anti-rabbit immunoglobulin G heavy and light chain Cy3 (Jackson Immunoresearch, 711-166-152; 1:200); fluorescein isothiocyanate (FITC)-conjugated mouse anti-PIMO as primary antibody (included in Hypoxyprobe™-1 kit; 1:50), with secondary detection of PIMO with goat anti-FITC (Abcam, ab19224, LOT GR175456-35; 1:100, incubated for 2 h at room temperature) followed by donkey anti-goat Alexa Fluor 488 (Jackson Immunoresearch, 705-546-147; 1:200).

Fluorescence images were recorded using a Leica SP8 confocal or a Leica DMR widefield microscope (equipped with bandpass filter blocks and a Jenoptik ProgRes MF USB camera). Complete tissue sections were digitized using a ZEISS Axioscan Z1 slide scanner.

### Image acquisition and processing

Complete tissue sections were digitized using a ZEISS Axioscan Z1 slide automated scanner for image acquisition of brightfield and multichannel fluorescence slides. During acquisition, specimens were autofocused, and individual fluorescence channels were recorded with identical settings [transmitted LED light source Colibri 7; 4′,6-diamidino-2-phenylindole (DAPI): Ex 385 nm, beam splitter 395 nm, Em bandpass 445/50 nm; Cy2: Ex 475 nm, beam splitter 495 nm, Em 525/50 nm; Cy3: Ex 555 nm, beam splitter 570 nm, Em 605/70 nm; Cy5: Ex 630 nm, beam splitter 660 nm, Em 690/50 nm] for all specimens. H&E-stained tissue specimens were recorded in brightfield. Multiple captured images were automatically tiled into a composite image of the entire organ by the ZEN software (ZEISS) and a representative image is presented in Fig. S3. From the image of the entire organ (Fig. S3), a lesion area is selected from each experimental group and presented in a higher magnification in Fig. S2C to depict the fluorescent intensity of each target examined.

### Bacterial enumeration from lungs and spleens

Mice were euthanized at dedicated time points and superior, middle inferior and post-caval lobes were removed and homogenized in 1 ml PBS/0.05% Tween 80. Serial dilutions of organ homogenates were plated onto Middlebrook 7H11 agar and, in addition, on agar supplemented with 0.4% activated charcoal for all time points during chemotherapy. Plates showing higher CFU counts were used for data analysis.

### Isolation of BMDMs

BMDMs were obtained from tibia and femur bones and maintained in Dulbecco's modified Eagle medium containing 20% L929-cell supernatant, 10% heat-inactivated fetal calf serum, 5% heat-inactivated horse serum, 2 mM glutamine. Differentiated resting cells and cells pretreated with recombinant mouse IFN-γ (100 U/ml; Strathmann Biotech AG) were infected with *M. tuberculosis* H37Rv at a multiplicity of infection of 1:5.

### Extracellular flux analysis

Seahorse XFp extracellular flux analyzer (Agilent, Santa Clara, CA, USA) was used to measure the OCR of *M. tuberculosis* cells as described earlier (Krishnamoorthy et al., 2019; Lamprecht et al., 2016). Seahorse XF96 extracellular flux analyzer (Agilent) was used to measure the OCR and extracellular acidification rate (ECAR) of murine BMDMs as per the manufacturer's recommendation. Cells were seeded into the XF96 cell culture plate at densities of 70,000 cells/well and rested for 24 h. Subsequently, cells were stimulated with IFN-γ (100 U/ml) for a further 24 h at 37°C/7% CO_2._ Mitochondrial respiration assay (Seahorse XF cell mito stress test) and glycolytic function assay (Seahorse XF glycolysis stress test) were performed according to the manufacturer's recommendation. Data analysis was carried out using Wave Desktop 2.6 Software (available at https://www.agilent.com/en/products/cell-analysis/software-download-for-wave-desktop) and the XF Report Generators for calculation of the parameters from the respective assays (Supplementary Materials and Methods).

## Supplementary Material

Supplementary information
