# Supplementary material for: FX11 limits Mycobacterium tuberculosis growth and potentiates bactericidal activity of isoniazid through host-directed activity
Source: Dis Model Mech. 2020 Mar 30;13(3):dmm041954. doi: 10.1242/dmm.041954 (PMC7132771; doi:10.1242/dmm.041954)
Supplement: Supplementary information [file dmm-13-041954-s1.pdf]

Supplementary information

Fig. S1

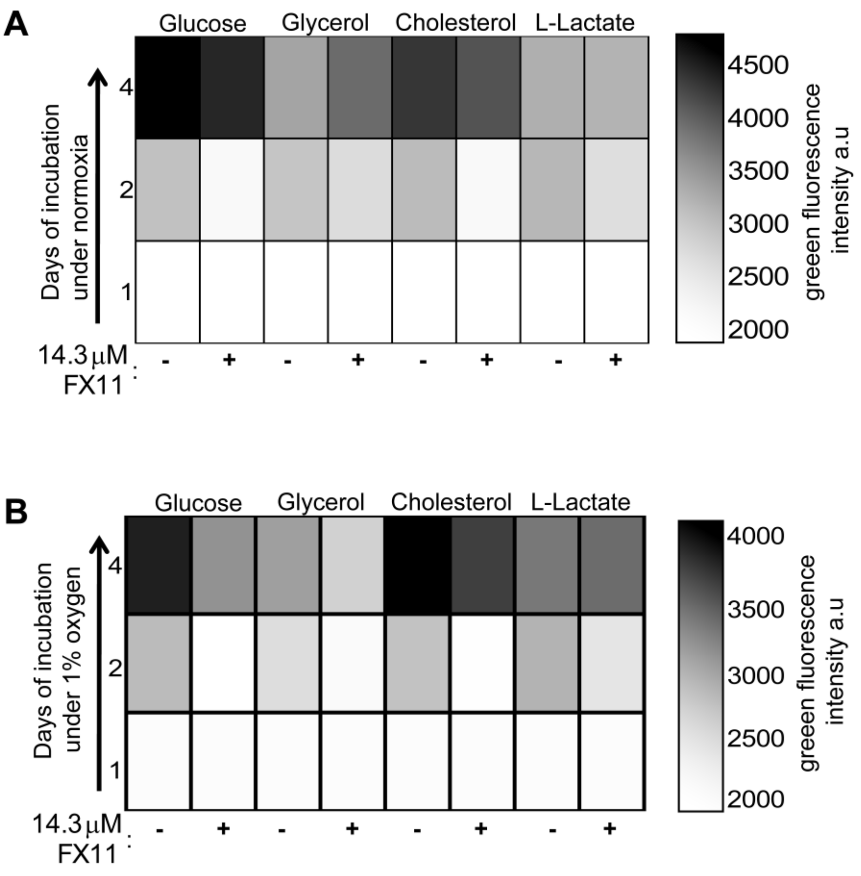

Fig. S1. Effects of FX11 on bacterial growth. (A-B) Gradient map showing the fluorescence intensity of green fluorescence protein expressing *M. tuberculosis* strain. Liquid culture in medium containing specified carbon sources and incubated under aerobic or hypoxic growth condition at 37 C for 4 days.

Disease Models & Mechanisms • Supplementary information

Fig. S2

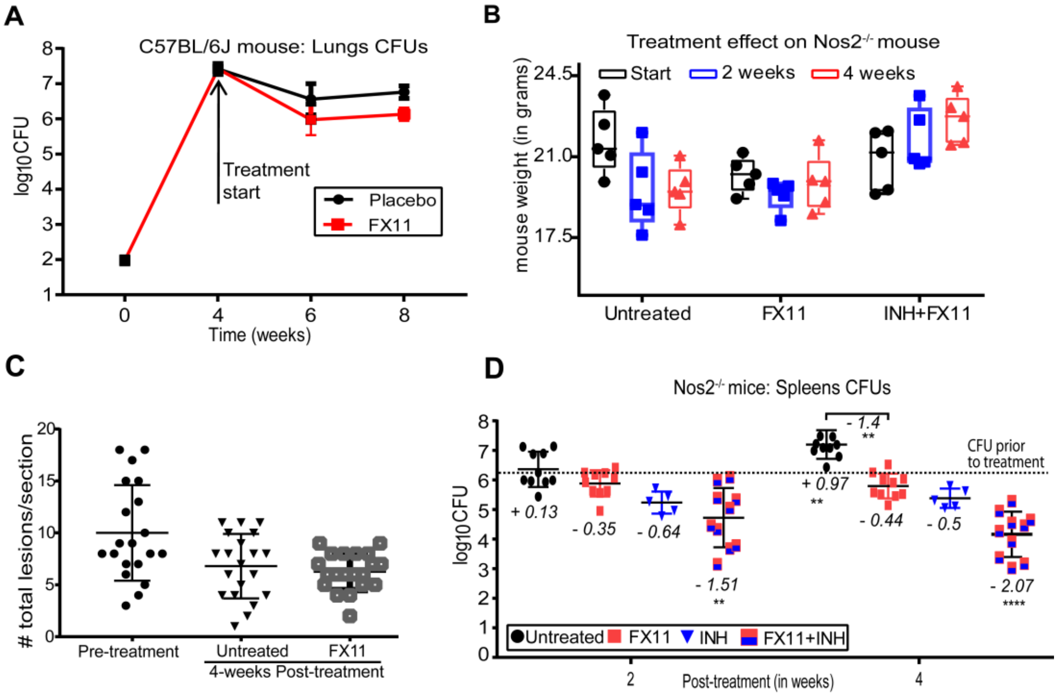

Fig. S2. Effects of FX11 on TB in mouse models. (A) Bacterial burden in C57BL/6J lungs (both placebo and FX11-treated group) are shown at respective time points (related to Fig. 2A). Datasets presented are from 2 independent experiments (total n = 10). Values shown are means±standard deviation (SD). **(B)** Body weight of untreated and drug-treated Nos2$^{-/-}$ mice. **(C)** Total number of lesions (necrotic and non-necrotic) per lung section of Nos2$^{-/-}$ mice groups. **(D)** Splenic CFU (means±SD) of untreated and drug-treated Nos2$^{-/-}$ mice. Splenic CFU counts (means±SD) from two independent experiments (total n = 9–10) are shown. In contrast, CFU data ((means±SD) of INH-treated group are from a single experiment with a group size of 5. Italicized numerical value (in negative) represents reduction or value (in positive) represents a further increase in $\log_{10}$CFU in the specified group, when compared with the control group prior to drug treatment (i.e. day 56, indicated in dotted line). Pooled data from two independent experiments were analyzed using nonparametric Mann-Whitney test (data that did not pass the Shapiro-Wilk normality test). Statistical significance as compared to the group prior to drug treatment, *p<0.05, **p<0.01, ****p<0.0001.

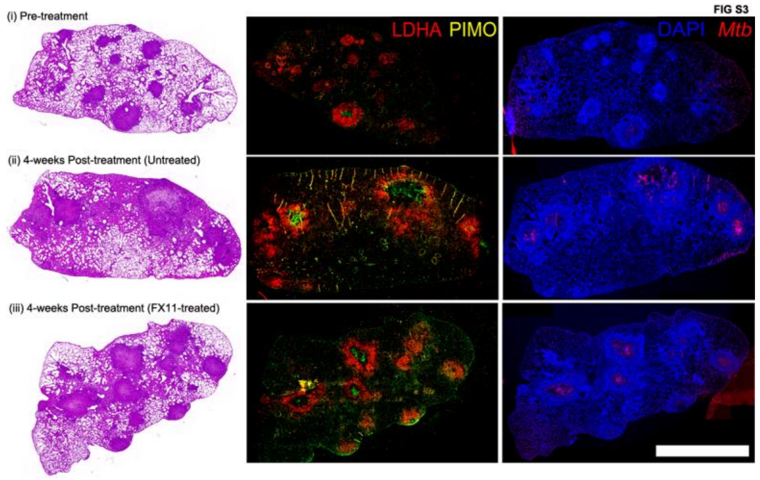

Fig. S3. Staining of whole lung section from Nos2$^{-/-}$ **mice.** Micrographs of stained consecutive thin sections of the fixed and paraffin-embedded left lung lobe. Scale bar represents 2.5 mm.

Disease Models & Mechanisms • Supplementary information

Supplementary Materials and Methods

Extracellular flux analysis: methodology and statistics.

Extracellular flux analysis. Seahorse Assay principle, design, and equations to calculate each of the parameters is schematically illustrated below using the representative data obtained in this study. A more detailed account of these assays can be accessed from the manufacturer's web resources or described in Cumming et al. 2018. (Cumming, B. M., Addicott, K. W., Adamson, J. H. and Steyn, A. J. (2018). *Mycobacterium tuberculosis* induces decelerated bioenergetic metabolism in human macrophages. eLife 7, e39169. https://doi.org/10.7554/eLife.39169).

Mitochondrial respiration

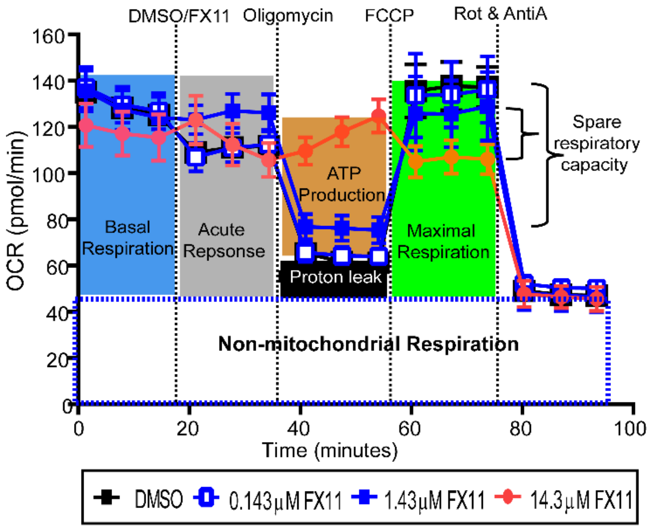

Parameter and rate measurement equation used

Non-mitochondrial Oxygen consumption	Minimum rate measurement after Rotenone/Antimycin A injection
Basal respiration	(Last rate measurement before first injection) – (Non-mitochondrial respiration rate)
Maximal Respiration	(Maximum rate measurement after FCCP injection) – (Non-mitochondrial respiration rate)
Proton leak	(Minimum rate measurement after Oligomycin injection) – (Non-mitochondrial respiration rate)
ATP Production	(Last rate measurement before Oligomycin injection) – (Minimum rate measurement after Oligomycin injection)
Spare Respiratory Capacity	(Maximal Respiration) – (Basal Respiration)

Glycolytic Function

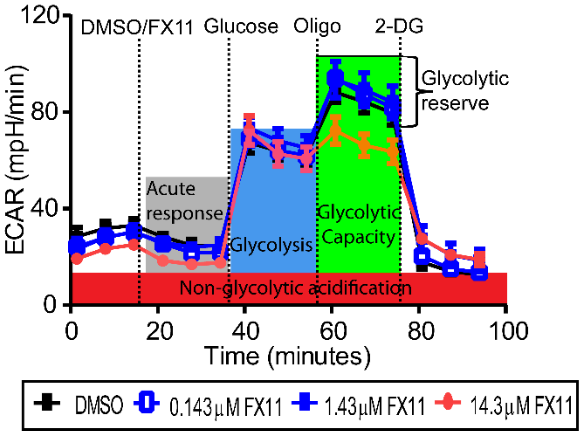

Parameter and rate measurement equation used

Glycolysis	(Maximum rate measurement before Oligomycin injection) – (Last rate measurement before Glucose injection)
Glycolytic Capacity	(Maximum rate measurement after Oligomycin injection) – (Last rate measurement before Glucose injection)
Glycolytic Reserve	(Glycolytic Capacity) – (Glycolysis)
Non-Glycolytic Acidification	Last rate measurement prior to glucose injection

Disease Models & Mechanisms • Supplementary information

Assay type, injection sequence of modulators used in this study.

Mitochondrial respiration assay:

	Mitochondrial respiration		
	**Compound**	**Function**	**Effect on OCR**
**Basal respiration**	Not applicable	-	To monitor the cellular energetic demand under baseline conditions.
**Injection 1**	DMSO or FX11	Lactate dehydrogenase A inhibitor solubilized in DMSO.	Test compound
**Injection 2**	Oligomycin mixture	Inhibitor of ATP synthase V of Electron transport chain	A decrease in OCR correlates cellular ATP generation to with mitochondrial respiration.
**Injection 3**	Carbonyl cyanide-4 (trifluoromethoxy) phenylhydrazone (FCCP)	Uncoupling agent results in maximum oxygen consumption rate by collapsing inner mitochondrial membrane.	An increase in OCR levels indicates maximum respiration capacity of cell.
**Injection 4**	Rotenone and antimycin A	Inhibitor of complexes I and III of electron transport chain	A decrease in OCR correlates with shut down of mitochondrial respiration. Cellular respiration is driven by non-mitochondrial process.

Disease Models & Mechanisms • Supplementary information

Glycolytic stress assay: Glucose is converted to pyruvate, and subsequently to lactate, results in proton generation and extrusion that acidify the extracellular medium (recorded as ECAR). This test was carried out to determine the impact of FX11 on ECAR values of BMDMs when sequentially treated with different glycolytic modulators.

	**Glycolytic stress**		
	**compound**	**function**	**effect on ECAR**
**Basal acidification**	Not applicable	-	Base line reading to assess non-glycolytic acidification
**Injection 1**	DMSO or FX11	Lactate dehydrogenase A inhibitor solubilized in DMSO.	Test compound
**Injection 2**	Glucose	Glycolytic substrate. Glucose catabolism result in pyruvate and lactate and subsequent extracellular release of protons	An increase in ECAR value correlates with rate of glycolysis
**Injection 3**	Oligomycin mixture	Inhibitor of mitochondrial ATP synthase. Upon inhibition, cells are increasingly dependent on glycolysis.	Further increase in ECAR value correlates with maximum glycolytic capacity of the cell (in the absence of oxidative phosphorylation).
**Injection 4**	2-deoxyglucose (2-DG)	Inhibitor of glucose hexokinase which mediates first step of glycolysis	A decrease in ECAR value implies that the ECAR produced in the experiment is due to glycolysis.

Data acquisition: Oxygen consumption (OCR) and extracellular acidification rates (ECAR) were measured using the Seahorse XF96 extracellular flux analyzer (Agilent, Santa Clara, CA). Two different assays were performed using the XF96: mitochondrial respiration assay, and glycolytic stress assay. Acquired real-time data were into the XF Report Generators using the Wave Desktop 2.6 software for calculation of the parameters from the specific assays.

Statistical analysis to determine the effect of FX11 on bone marrow derived macrophages bioenergetics and glycolytic response.

Data acquisition: Oxygen consumption (OCR) and extracellular acidification rates (ECAR) were measured using the Seahorse XF96 extracellular flux analyzer (Agilent, Santa Clara, CA). Two different assays were performed using the XF96: mitochondrial respiration assay, and glycolytic stress assay. Acquired real-time data were into the XF Report Generators using the Wave Desktop 2.6 software for calculation of the parameters from the specific assays. The effect of FX11 was statistically analyzed using either t-test or linear regression modelling.

Results:

1. **Statistical analysis of calculated respiratory parameters of BMDMs treated with FX11 or DMSO (control).**

1.1. Box plots showing respiratory response (OCR value) stratified by experiment replicate (related to Fig. 1A and B).

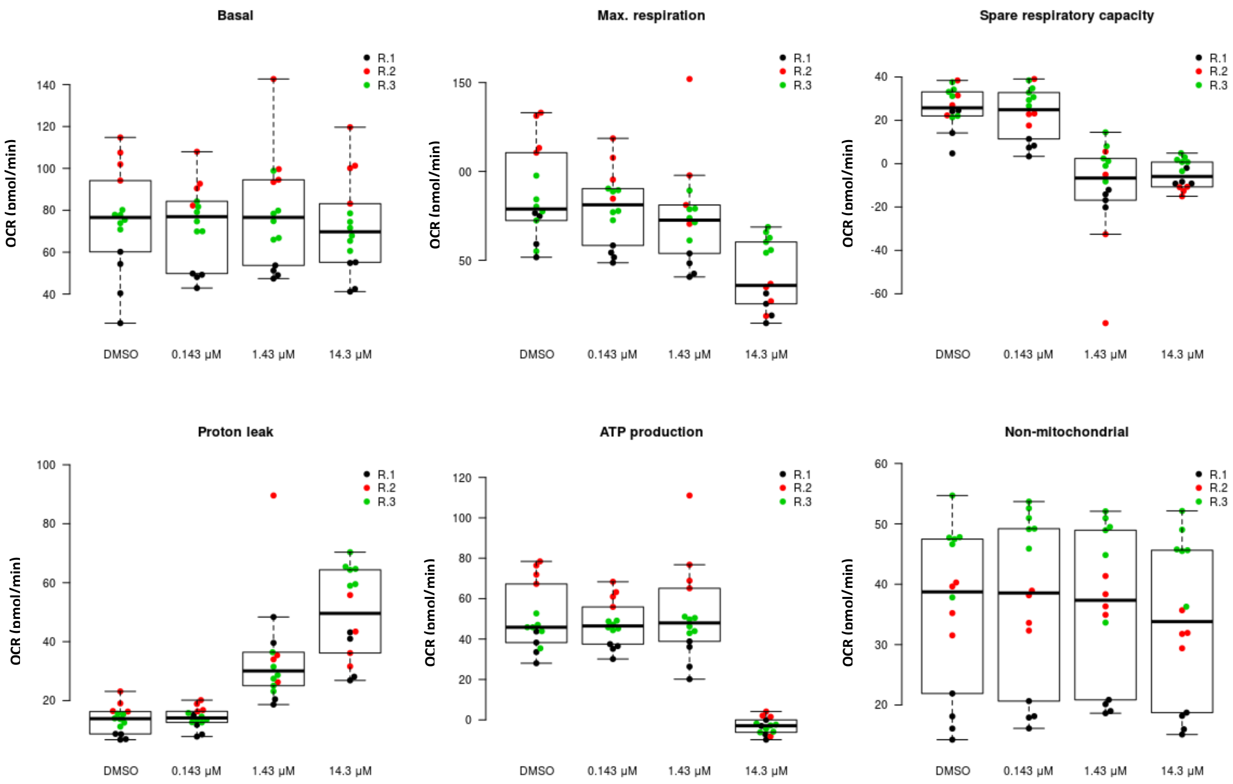

Disease Models & Mechanisms • Supplementary information

1.2. Results of t-test for each of the concentration, as compared to the DMSO control. *p*, p-value from Welch t-test; *p.adj*, p-value corrected for multiple testing using the Benjamini-Hochberg correction; *d*, Cohen's d estimate of effect size. For effect size, *d > 0.5* is considered a medium effect, and *d > 0.8* is considered a large effect.

**Respiratory parameters (related to data presented in Fig. 1B)**	FX11 Concentration (in µM)	d	p	p.adj
Basal	0.143	-0.27	0.48	0.59
	1.43	0.25	0.510	0.59
	14.3	-0.30	0.43	0.59
Max. respiration	0.143	-0.52	0.19	0.36
	1.43	-0.68	0.08	0.21
	14.3	-2.36	1.4e-06	6.5e-06
Spare respiratory capacity	0.143	-0.35	0.36	0.55
	1.43 µM	-2.47	3.6e-06	1.28e-05
	14.3 µM	-4.44	2.2e-10	2.0e-09
Proton leak	0.143 µM	0.25	0.52	0.56
	1.43 µM	1.68	0.00058	0.0018
	14.3 µM	3.74	1.061e-07	6.40e-07
ATP production	0.143 µM	-0.54	0.17	0.36
	1.43 µM	0.13	0.74	0.74
	14.3 µM	-6.09	1.74e-13	3.13e-12
Non-mitochondrial	0.143 µM	0.36	0.35	0.55
	1.43 µM	0.17	0.66	0.70
	14.3 µM	-0.50	0.20	0.36

1.3. Linear regression models for each of the six respiratory parameters (see above box plots in 1.1)

For each parameter (readout), the influence of FX11 concentration on the parameter readout was tested using log-linear regression. To this end, the FX11 concentrations were logarithmized (with the control, DMSO, assumed to have a concentration below 0.0143 mM) and a linear model (lm) was fit on the resulting data with the lm() function in R.

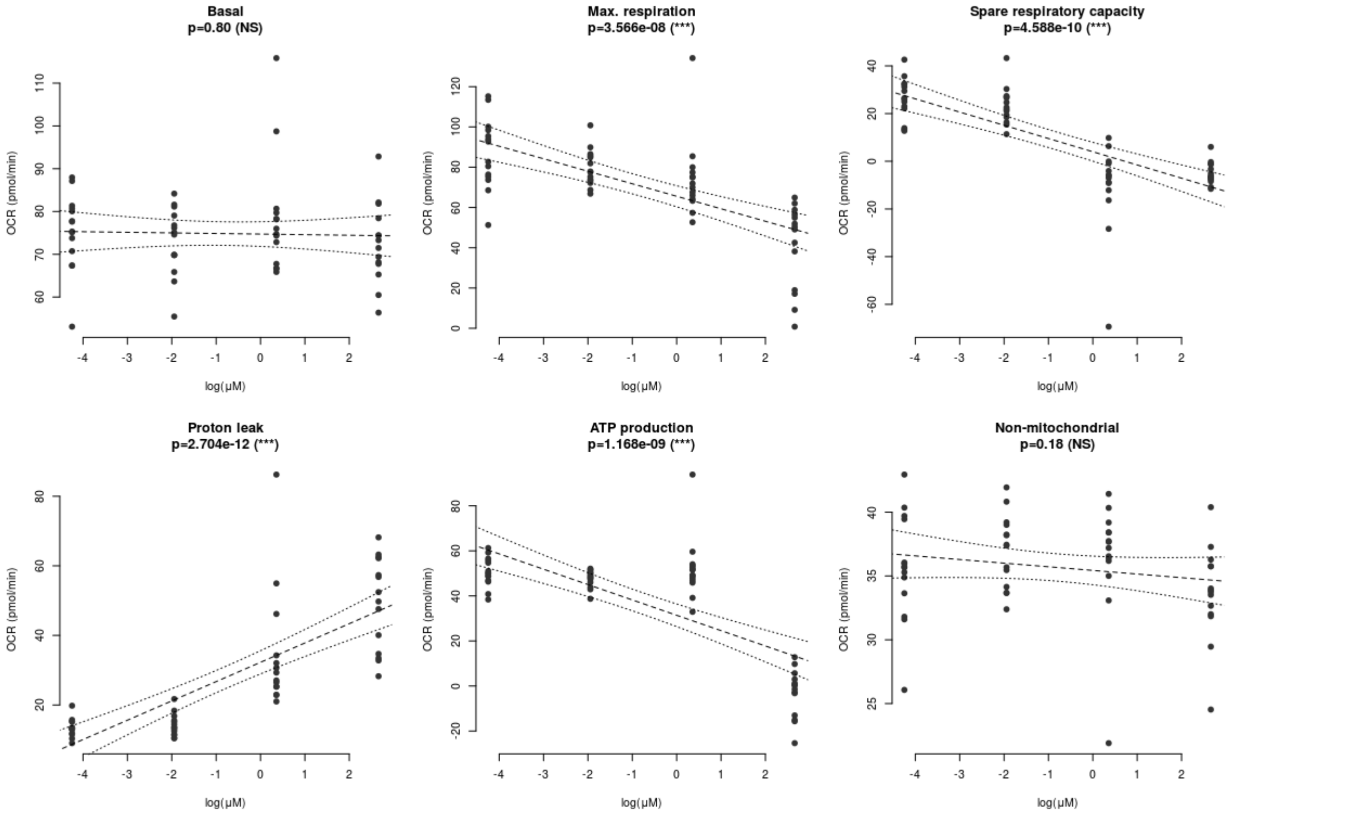

Linear regression modeling results (related to data presented in Fig. 1B).

Parameter	P values
Basal	0.80 (Not significant)
Max. respiration	6.4e-08
Spare respiratory capacity	9.70e-10
Proton leak	6.86e-12
ATP production	2.38e-09
Non-mitochondrial	0.18 (Not significant)

2. Glycolytic stress profile and glycolytic parameters of BMDMs-treated with FX11 or DMSO (vehicle control).

2.1. Box plots showing glycolytic response (ECAR value) stratified by experiment replicate (related to Fig. 1C and D).

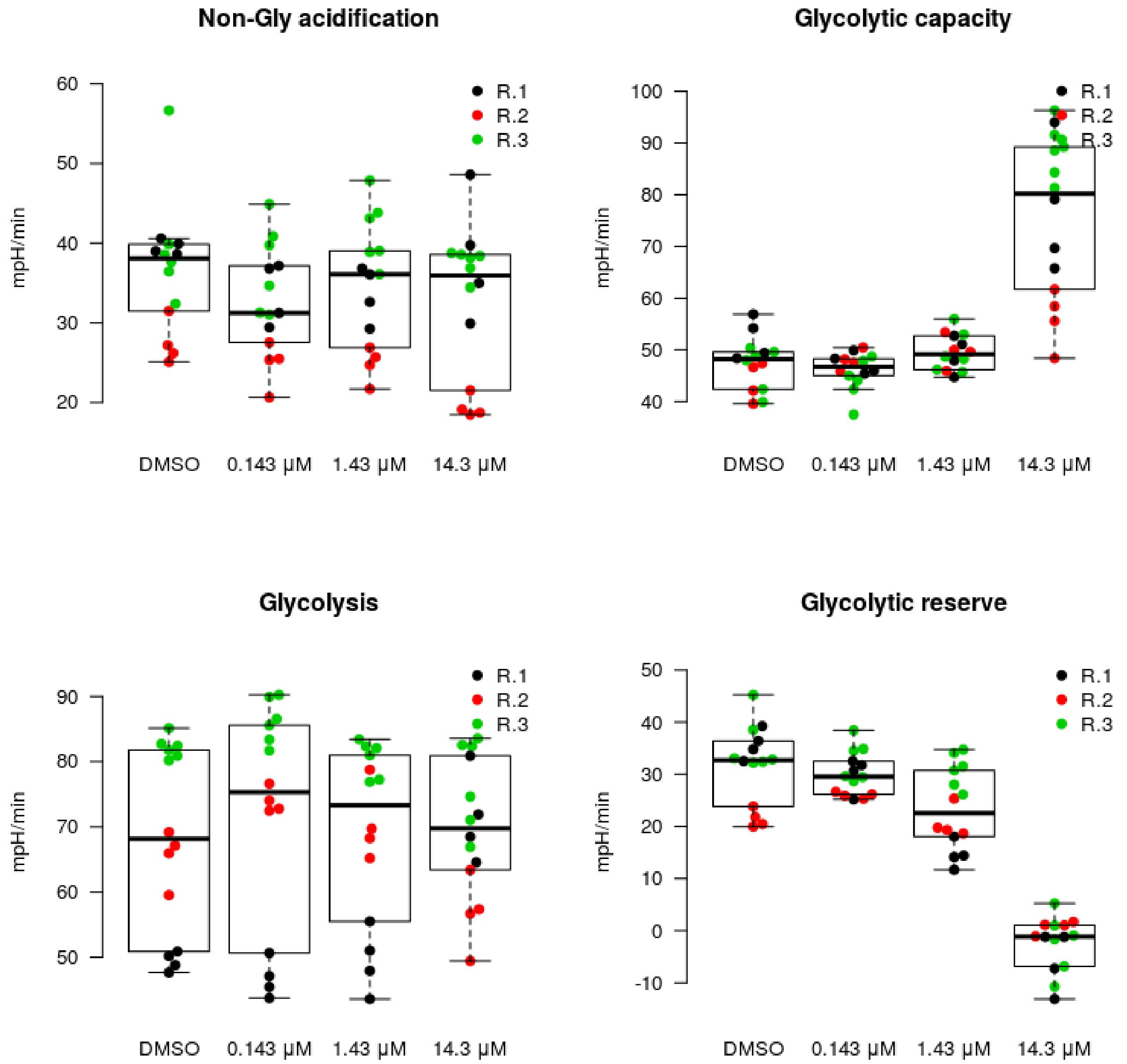

2.2. Glycolytic parameters: Results of t-test for each of the concentration, as compared to the DMSO control. *p*, p-value from Welch t-test; *p.adj*, p-value corrected for multiple testing using the Benjamini-Hochberg correction; *d*, Cohen's d estimate of effect size. For effect size, *d > 0.5* is considered a medium effect, and *d > 0.8* is considered a large effect.

**Glycolytic parameters (Fig. 1B)**	FX11 Concentration (in µM)	d	p	*p.adj*
Non-Gly acidification	0.143	-0.76	0.054	0.15
	1.43	-0.40	0.3	0.45
	14.3	-0.74	0.06	0.15
Glycolytic capacity	0.143	-0.22	0.56	0.68
	1.43	0.43	0.26	0.45
	14.3	2.9	6.7e-07	4.04e-06
Glycolysis	0.143	0.63	0.11	0.23
	1.43	0.17	0.66	0.68
	14.3	0.16	0.68	0.68
Glycolytic reserve	0.143	-0.34	0.38	0.50
	1.43	-1.43	0.00086	0.0034
	14.3	-5.46	6.3e-14	7.5e-13

Disease Models & Mechanisms • Supplementary information

2.3. Linear regression models for each of the four outputs (see above box plots in 2.1.)

For each parameter (readout), the influence of FX11 concentration on the parameter readout was tested using log-linear regression. To this end, the FX11 concentrations were logarithmized (with the control, DMSO, assumed to have a concentration below 0.0143 mM) and a linear model (lm) was fit on the resulting data with the lm() function in R.

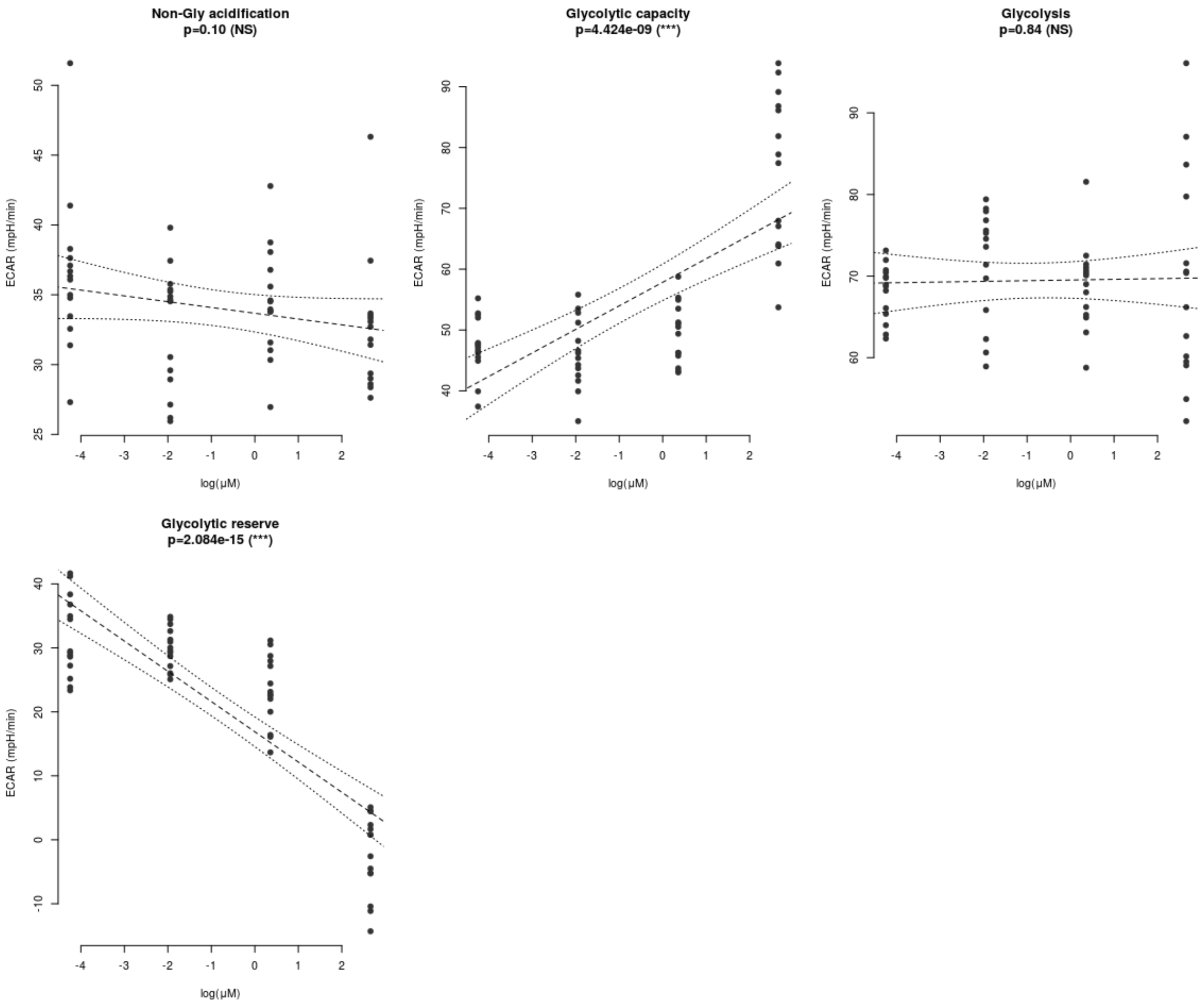

Linear regression modeling results (related to glycolytic function parameters presented in Fig. 1C and D).

Parameter	P values
Non-Gly acidification	0.11 (Not significant)
Glycolytic capacity	8.57e-09
Glycolysis	0.85 (Not significant)
Glycolytic reserve	6.88e-15

Disease Models & Mechanisms • Supplementary information
